# Effects of Deep Sedation With Dexmedetomidine Versus Remifentanil on Postoperative Recovery in Soft Tissue Surgery

**DOI:** 10.7759/cureus.79820

**Published:** 2025-02-28

**Authors:** Aleksandar M Kishman, Marija V Sholjakova, Andrijan Kartalov, Biljana Kuzmanovska, Albert Lleshi, Marija Jovanovski Srceva, Vesna Durnev

**Affiliations:** 1 Anesthesiology and Critical Care, University Clinic for Traumatology, Orthopedics, Anesthesia and Intensive Care and Emergency, Faculty of Medicine, Saints Cyril and Methodius University, Skopje, MKD

**Keywords:** analgesia and sedation, dexmedetomidine, pain, postoperative recovery, qor-15, remifentanil

## Abstract

Background

Soft tissue surgery comprises short or medium-duration surgical procedures, with anesthesia consisting of analgesia and sedation. Various quantitative and qualitative recovery scales are used to evaluate the quality of postoperative recovery. The primary objective of this study was to compare the effects of dexmedetomidine versus remifentanil on postoperative recovery using the Quality of Recovery-15 (QoR-15) scale to assess recovery quality in soft tissue surgeries.

Methodology

This prospective randomized study was conducted at the Clinic of Anesthesia, Reanimation and Intensive Care and University Clinic of Reconstructive and Plastic Surgery, Skopje, Republic of North Macedonia, involving 80 patients. Patients were randomly assigned into two groups, namely, Group 1, sedated with intraoperative dexmedetomidine infusion (Dex, n = 40), and Group 2, sedated with intraoperative remifentanil infusion (Rem, n = 40). Intraoperatively, hemodynamic and respiratory parameters were measured. Preoperatively and postoperatively, levels of leukocytes, blood sugar, and the QoR-15 score were determined. Postoperative mean arterial pressure (MAP), postoperative pain (Visual Analog Scale), and sedation level (Richmond Agitation-Sedation Scale) were compared with the quality of postoperative recovery scores (QoR-15) using Pearson’s correlation coefficient.

Results

The results indicated that dexmedetomidine provided prolonged postoperative sedation and analgesia, which dissipated shortly thereafter, while patients sedated with remifentanil experienced pain immediately upon awakening and required more analgesics. The correlation analysis showed a negative relationship between the degree of postoperative pain and sedation and the quality of recovery.

Conclusions

Dexmedetomidine demonstrated a superior performance compared to remifentanil. Hence, dexmedetomidine in soft tissue surgery ensures hemodynamic stability, shows protective anti-inflammatory and anti-stress effects, provides good postoperative analgesic effects, reduces recovery time, and protects the body from undesirable postoperative complications.

## Introduction

Soft tissue surgery encompasses a wide range of procedures performed on the body’s surface and soft tissues (skin, muscles, and subcutaneous fat) without affecting the bones, periosteum, internal linings, or organs. Although classified as minor surgery, it presents its challenges. Pain in these regions is often intense, and patients tend to be anxious, scared, and restless, which can impact the surgical atmosphere. An anesthesiologist should decide between general anesthesia and deep sedation with analgesia, with the latter ensuring a safe postoperative course given the nature of the surgery, which typically requires rapid discharge (day surgery).

For this purpose, drugs for intraoperative sedation and analgesia appear to meet the requirements for a calm, pain-free patient during soft tissue surgery. In practice, several agents used alone or in combination are advised in the literature for intraoperative sedation and analgesia. The most used are midazolam or propofol, often combined with fentanyl for analgesia [1].

The properties of dexmedetomidine as a sedative, anxiolytic, and analgesic and remifentanil’s ability to provide effective analgesia-sedation at low doses suggest that these two agents are suitable for use in soft tissue surgery.

Dexmedetomidine is a highly selective alpha-2 adrenergic receptor agonist in the central nervous system. Its sedative effects result from inhibiting neuronal transmission in the locus coeruleus (LC) in the brainstem, while its analgesic effect is attributed to direct central action on the LC [2]. The sympatholytic effect of dexmedetomidine induces hemodynamic changes [3]. Combined with benzodiazepines, opioids (e.g., remifentanil), or propofol, dexmedetomidine enhances sedation, helps maintain hemodynamic stability, and reduces the need for other sedatives [4]. It also influences the endocrine system by decreasing catecholamine secretion and the sympathetic stress response, resulting in an anti-stress effect. This, along with its associated effects, natural awakening, and patient satisfaction, makes it a captivating agent in anesthesiology [5]. It is administered as a bolus infusion over the first 10 minutes, followed by continuous infusion.

Remifentanil is a synthetic opioid and a mu-receptor agonist. It is a potent analgesic that reduces sympathetic nervous system tone and, similar to all opioids, causes respiratory depression. Its rapid onset and short recovery time make it appealing for use in soft tissue surgery, where it is administered as a low-dose infusion for hypnosis and sedation [6].

The decision to be safe for patient discharge following analgesia-sedation is significant. Therefore, the level of recovery after anesthesia and surgery must be evaluated. In practice, various scales are used to assess pain, sedation, or satisfaction levels, such as the Visual Analog Scale (VAS) for pain, the Ramsay Sedation Scale (RSS) for sedation, and scales for anesthesia satisfaction, among others [7,8]. The Quality of Recovery Score-40 (QoR-40) and the modified version QoR-15 measure the quality of recovery following anesthesia or analgesia-sedation [9,10]. These are comprehensive scales assessing the outcomes of sedation, anesthesia, and surgery.

The original QoR-40 postoperative recovery quality scale is a multidimensional tool developed by Myles et al. in 2000 [9,11]. It includes a specific questionnaire with 40 items measuring the following five dimensions: physical comfort (12 items), emotional state (nine items), physical independence (five items), psychological support (seven items), and pain (seven items) [12,13]. While this scale provides detailed measurement of postoperative conditions, its modified version, the QoR-15 scale, developed by Stark et al. (2013), is increasingly preferred for its practicality, requiring just a few minutes to administer [10,14,15]. This questionnaire is divided into the following two parts: section 1 provides the evaluation of physical condition of the patient (10 questions scored from 0 to 10; 0 = very poor, and 10 = excellent postoperative recovery quality); and section 2 presents the mental condition of the patient including pain evaluation. It has five questions scored from 10 to 0 (10 = feeling much, and 0 = not at all). The usefulness and value of this scale has been proven by multiple studies confirming that it represents a comprehensive, patient-oriented measure for recovery after surgery [14-16].

The absence of studies evaluating how and to what extent sedative-analgesic agents contribute to post-anesthetic and surgical recovery, as well as questions regarding their effect on outcomes, present a challenge for every anesthesiologist. The need to address these questions led to this study. To assess the quality of postoperative recovery with the use of dexmedetomidine or remifentanil for sedation/analgesia and to determine which perioperative and postoperative factors influence recovery, a comparative analysis was conducted to correlate these variables with the outcome measure QoR-15.

## Materials and methods

This prospective, randomized clinical study was conducted at the University Clinic for Anesthesia, Reanimation, and Intensive Care (CARIC), part of the University Clinic for Traumatology, Orthopedics, Anesthesia and Intensive Care and Emergency Center at the University Clinical Center “Mother Theresa” at the Faculty of Medicine in Skopje, in collaboration with the University Clinic for Plastic and Reconstructive Surgery (UC PRS), from May to October 2024.

This prospective study received approval from the Institutional Ethics Committee. After obtaining written consent, 80 patients who met the inclusion criteria were enrolled in the study. The inclusion criteria were as follows: patients admitted for soft tissue surgical procedures, aged >25 years, with a body mass index (BMI) <35 kg/m², classified as American Society of Anesthesiologists (ASA) Physical Status I-III, who were alert, oriented, and communicative, and who provided written consent to participate in the study. We excluded patients who did not meet the inclusion criteria, had severe cardiovascular diseases (serious heart disease, heart block or atrioventricular block, cardiac arrhythmias, low arterial blood pressure due to dehydration or bleeding, or very high blood pressure), or declined to participate in the study.

Patients were randomly divided into two groups. Group 1 consisted of patients who received a perioperative infusion of dexmedetomidine (Dex) (n = 40), while Group 2 received a remifentanil (Rem) infusion (n = 40). All participants were prepared as outpatients and informed about the study methods for pain assessment (VAS scale) and postoperative recovery evaluation (QoR-15 scale). Additionally, a full blood sample was collected for routine laboratory tests, leukocytes, and blood glucose analysis.

One hour before the intervention, patients received oral premedication with either midazolam 1 mg or diazepam 2.5 mg. Before entering the operating room, they were surveyed using the QoR-15 questionnaire, and continuous hemodynamic and respiratory monitoring was initiated.

For the randomized group receiving dexmedetomidine (n = 40), sedation was induced with an initial bolus dose of 0.9 µg/kg of dexmedetomidine administered for 10 minutes, followed by a maintenance dose of 0.5 µg/kg/hour. In the remifentanil group (n = 40), sedation was induced with a dose of 0.09 µg/kg/minute of remifentanil until patients fell asleep, after which the dose was gradually reduced to 0.05 µg/kg/minute to provide an analgesic effect without impairing respiration.

Intraoperatively, all participants in the study were maintained for safe anesthesia with optimal disturbances of perioperative hemodynamic and respiratory values. Non-invasive methods were used for continuous perioperative monitoring, including electrocardiography (ECG), mean arterial pressure (MAP), heart rate at beats per minute (bpm), blood oxygen saturation percentage (SpO_2_), and the amount of end-tidal carbon dioxide (EtCO_2_). These parameters were recorded at five times during the sedation at 5, 10, 15, and 25 minutes after the start of surgery, as well as at the end of the surgery.

Preoperatively and postoperatively, blood samples were taken to monitor basic markers of inflammation and stress (white blood cell (WBC) count, C-reactive protein (CRP), blood glucose), and the quality of postoperative recovery was assessed using the QoR-15 scale.

Postoperative complications such as shivering, nausea, vomiting, postoperative pain (VAS), and sedation level (RSS) were recorded in four postoperative time intervals, namely, T0 (immediately after awakening), T1 (30 minutes after awakening), T2 (60 minutes after awakening), and T3 (120 minutes after awakening). These findings were compared with the QoR-15 scores obtained for assessing the quality of postoperative recovery.

The results were statistically processed using Microsoft Excel 2011 (Microsoft Corp., Redmond, WA, USA). Average and standard deviation were calculated, and statistically significant differences were assessed using the t-test. Statistical analysis of the correlation between postoperative parameters and the quality of recovery scale was performed using Pearson’s correlation test.

## Results

**Figure 1:**
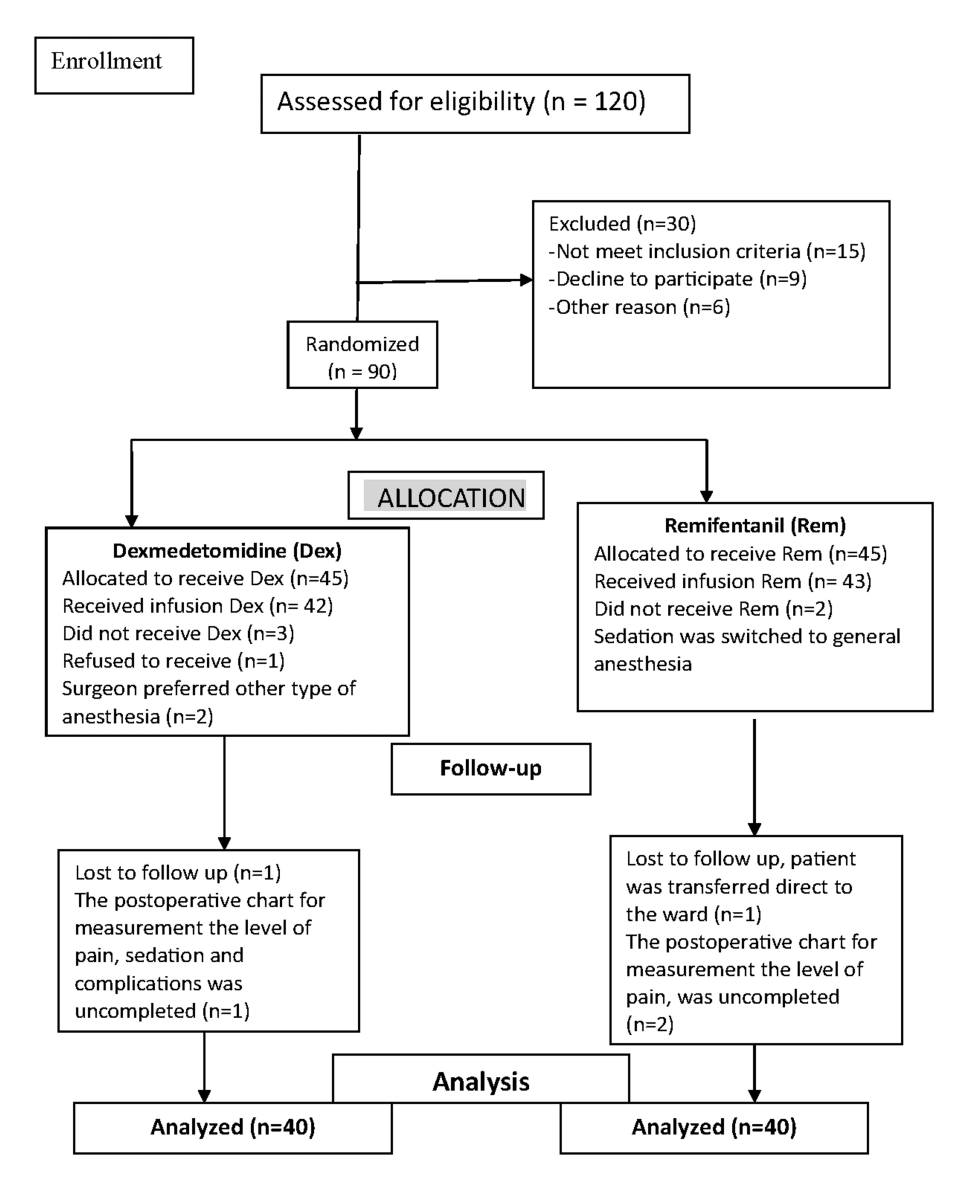
CONSORT flow diagram.

The study included 80 hospitalized patients who underwent soft tissue surgery (Figure 1). Patients were randomly assigned into two groups, each consisting of 40 patients.

To determine the homogeneity between the groups, an analysis of the demographic data of all participants was performed (age, gender, body weight, height, ASA classification, education level, and duration of anesthesia) (Table 1). The average age of the patients in the dexmedetomidine group was 70.633 ± 10.60 years, while the average age of the patients in the remifentanil group was 59.95 ± 12.52 years. The difference in the average age between the two groups was found to be statistically significant (p < 0.05), while no significant differences were observed in the other parameters (p > 0.05) (Table 1).

**Table 1:** Demographic characteristics of the participants in both groups. Data are presented as mean ± SD and N (%); t = Student’s t-test; p-values <0.05 were considered significant. ASA = American Society of Anesthesiologists

Variables	Dexmedetomidine (n = 40)	Remifentanil (n = 40)	t	P-value
Age (years), mean ± SD	70.633 ± 10.60	59.95 ± 12.52	0.037923	0.969864
Body weight (kg), mean ± SD	76.85 ± 10.42	77.05 ± 11.06	0.468141	0.641018
Body height (cm), mean ± SD	170.4 ± 8.59	172.825 ± 9.54	0.123822	0.523395
ASA I, n (%)	3 (4.5%)	9 (22.5%)	N/A	N/A
ASA II, n (%)	29 (72.5%)	25(62.5%)	N/A	N/A
ASA III, n (%)	8 (20%)	6 (15%)	N/A	N/A
Sex, male/female, n (%)	22/18 (55%/45%)	18/22 (45%/55%)	N/A	N/A
Primary school, n (%)	2 (5%)	3 (4.5%)	N/A	N/A
Secondary school, n (%)	19 (47.5%)	17 (42.5%)	N/A	N/A
Higher education, n (%)	19 (47.5%)	20 (50%)	N/A	N/A
Duration of anesthesia (minutes), mean ± SD	48.205 ± 11.726	50.5 ± 22.3803	0.314446	0.754057

Table 2 presents the values of the perioperative hemodynamic variables, MAP and pulse, measured at the following four time points: MAP 0 pre-operation, MAP 1-5 minutes post-incision, MAP 2-10 minutes post- incision, MAP 3-15 minutes post-incision, MAP 4-25 minutes post-incision, and MAP 5 end of operation.

**Table 2:** Perioperative hemodynamic variables of MAP and pulse (beats/minute) in the groups. Data are presented as mean ± SD MAP 0 = preoperative; MAP 1 = five minutes of surgery; MAP 2 = 10 minutes surgery; MAP 3 = 15 minutes surgery; MAP 4 = 25 minutes surgery; MAP 5 = end of surgery. MAP = mean arterial pressure

MAP (mmHg)	Dexmedetomidine (n = 40)	Remifentanil (n = 40)	t-	P-value
MAP 0	115.47 ± 13.47	107.125 ± 12.07	0.002	0.998409
MAP 1	106.77 ± 12.48	97.39 ± 10.58	0.000343	0.999761
MAP 2	95.82 ± 14.86	88.25 ± 11.72	0.007894	0.993796
MAP 3	88.8 ± 16.19	82.65 ± 13.40	0.037512	0.970182
MAP 4	82.95 ± 16.39	81.07 ± 12.17	0.286392	0.775407
MAP 5	93.2 ± 14.22	85.22 ± 11.64	0.004541	0.996421
Pulse (beats/minute)				
Preoperative	81.22 ± 19.10	88.5 ± 16.129	0.038281	0.969626
5 minutes after induction	77.77 ± 1.89	82.4 ± 14.134	0.090701	0.927963
10 minutes after induction	73.07 ± 11.89	78.3 ± 13.529	0.038281	0.969626
15 minutes after induction	72.6 ± 11.26	74.3 ± 12.724	0.269492	0.788333
25 minutes after induction	71.35 ± 11.26	73.975 ± 11.591	0.148604	0.882253
End of surgery	74.35 ± 12.30	75.775 ± 11.419	0.301048	0.764215

A significant decrease in MAP compared to preoperative pressure (MAP 0:MAP 1) was noted in both the dexmedetomidine and remifentanil groups (p = 0.00035 and p = 0.004567, respectively), as well as a significant difference in MAP 1, 2, and 5 between the groups (p < 0.05) and no significant difference at 15 and 25 minutes after surgery (p > 0.05). Regarding pulse frequency (bpm), a decrease was observed with a significant difference between preoperative pulse values and after five minutes of administration (p < 0.05), with no significant intergroup differences (p > 0.05).

The perioperative respiratory rate (breaths per minute), blood oxygen saturation (SpO_2_) (%), and end-tidal carbon dioxide (EtCO_2_) values (mmHg) are presented in Table 3. During the measurement times, the respiratory frequency in the remifentanil group decreased, reaching the lower limit of normal values. A significant difference in frequency between the groups was noted at 10, 15, and 25 minutes, and up to the end of the surgery (p = 0.000757, p = 0.000298, p = 4.19058E-05, and p = 0.011692, respectively). Perioperatively, blood oxygen saturation in both groups remained within the normal range. Significant differences between the groups were noted in the preoperative values and five minutes after the start of the surgery. No intergroup differences in EtCO_2_ values were observed at any measurement time.

**Table 3:** Perioperative respiratory variables in the studied groups. Data are presented as mean ± SD. P-values <0.05 are considered significant. SpO_2_ = blood oxygen saturation; EtCO_2_ = end-tidal CO_2_

Time	Dexmedetomidine (n = 40)	Remifentanil (n = 40)	t	P-value
Preoperative	14.6 ± 2.747	13.7 ± 2.587	0.072664	0.94231
5 minutes of surgery	13.975 ± 1.981	13.275 ± 2.368	0.082993	0.934143
10 minutes of surgery	14.15 ± 1.868	12.675 ± 2.040	0.000757	0.999443
15 minutes of surgery	13.625 ± 1.870	12.3 ± 1.310	0.000298	0.048981
25 minutes of surgery	13.375 ± 1.1776	11.9 ± 1.284	4.19058E-05	0.000073
End of surgery	13.015 ± 1.969	12.175 ± 1.123	0.011692	0.990774
SpO_2_ (%)				
Preoperative	97.45 ± 1.681	99 ± 1.306	1.21427E-05	0.228335
5 minutes of surgery	98.4 ± 1.634	98.95 ± 1.146	0.046662	0.962951
10 minutes of surgery	98.55 ± 1.378	99.125 ± 0.915	0.038281	0.969626
15 minutes of surgery	98.95 ± 1.324	98.925 ± 0.972	0.462731	0.644868
25 minutes of surgery	98.925 ± 1.386	98.925 ± 1.021	0.5	0 .618483
End of surgery	98.65 ± 1.279	98.6 ± 2.173	0.451484	0.652954
EtCO_2_ (mmHg)				
Preoperative	31.85 ± 2.363	33.65 ± 10.31	0.14863	0.882253
5 minutes after surgery	31.725 ± 1.912	31.45 ± 2.208	0.281564	0.779072
10 minutes after surgery	31.525 ± 1.912	30.9 ± 2.573	0.281564	0.779072
15 minutes after surgery	31.2 ± 1.823	30.15 ± 2.504	0.281564	0.779072
25 minutes after surgery	31.722 ± 1.856	31.55 ± 2.954	0.019909	0.984174
End of surgery	31.625 ± 2.702	31.91667 ± 1.331	0.385481	0.70099

As shown in Table 4, there was no significant difference in the preoperative leukocyte count between the dexmedetomidine and remifentanil groups (p = 0.498091), as well as in the postoperative count (p = 0.410673). Blood glucose showed a significant difference between the groups in both preoperative and postoperative periods (p = 0.046 and p = 0.002, respectively). This increase was statistically insignificant in the dexmedetomidine group (p = 0.069), and highly significant in the remifentanil group (p < 0.05).

**Table 4:** Preoperative and postoperative values for basic markers of inflammation, stress, and recovery score (QoR-15). P-values <0.05 are considered significant. QoR-15 = Quality of Recovery-15

Variable	Preoperative		t-test (p-value)	Postoperative		t-test (p-value)
	Dexmedetomidine (n = 40)	Remifentanil (n = 40)		Dexmedetomidine (n = 40)	Remifentanil (n = 40)	
Leukocytes (×10^9^/L)	7.43 ± 2.91	7.61 ± 2.26	0.498091 (p > 0.05)	7.38 ± 2.861	7.61 ± 2.27	0.410673 (p < 0.05)
Blood glucose (mmol/L)	5.77 ± 0.90	6.15 ± 1.039	0.046621 (p > 0.05)	6.64 ± 0.63	7.16 ± 0.73	0.002102 (p > 0.05)
Blood glucose, dexmedetomidine	5.77 ± 0.90		N/A	6.64 ± 0.63		0.0694 (p = 0.94)
Blood glucose, remifentanil	6.15 ± 1.039		N/A	7.16 ± 0.73		3.22422E-06 (p = 0.00184)
QoR-15 score	126.41 ± 7.6	137.4 ± 3.71	t = 1.09, p = 0.279	92.72 ± 32	95.77 ± 32.7	t = 0.3424, p = 0.7649

The results of preoperative and postoperative values for recovery score (QoR-15), level of pain (VAS), and sedation scores (RSS) are presented in Table 5. Postoperative pain was measured using the VAS at the following four time points: VAS 0 (immediately after awakening), VAS 1 (30 minutes after awakening), VAS 2 (60 minutes after awakening), and VAS 3 (120 minutes after awakening). The values are displayed in Table 5, along with the sedation scores measured on the RSS at the same time points. The comparison between the two groups showed significantly lower pain scores in the remifentanil group at VAS 0 and VAS 1 (1.7 ± 1.328 and 2.25 ± 0.573, respectively), and significantly higher pain scores at VAS 3 (4.425 ± 2.667). No significant changes in the VAS score were observed in the dexmedetomidine group between the different time points (p = 0.069429, p = 0.125184, and p = 0.075539, respectively). In the remifentanil group, a highly significant difference in pain levels was found across different time points. The measurements of the sedation level showed a statistically significant intergroup difference in RSS 0, RSS 2, and RSS 3 (p < 0.05) (Table 5).

**Table 5:** Postoperative values of pain (VAS) and sedation scores (RSS). Data are presented as mean ± SD; t = Student’s t-test values; p-values <0.05 are considered significant. VAS = Visual Analog Scale; RSS = Ramsay Sedation Scale

	Dexmedetomidine (n = 40)	t-test (p-value)	Remifentanil (n = 40)	P-value	t-test (p-value)
Pain scores					
VAS 0	2.475 ± 0.62	N/A	1.7 ± 1.328	N/A	5.65311E-06 (p < 0.00001)
VAS 1	2.025 ± 0.71	0.069429 (p = 0.944)	2.25 ± 0.573	0.000109	2.7558E-11 (p = 0.00728)
VAS 2	1.675 ± 0.71	0.125184 (0.9)	2.2 ± 0.395	0.000000	0.5 (p = 0.61848)
VAS 3	2.47 ± 0.62	0.07553 (0.94)	4.425 ± 2.66	0.000000	7.46219E-14 (p < 0.00001)
Sedation scores					
RSS 0	1.875 ± 0.45	N/A	2.70 ± 0.592	N/A	0.001225 (p = 0.9990)
RSS 1	2.025 ± 0.15	N/A	2.37 ± 0.817	N/A	0.113825 (p = 0.9096)
RSS 2	1.975 ± 0.15	N/A	2.25 ± 0.757	N/A	0.000581 (p = 999602)
RSS 3	1.95 ± 0.308	N/A	1.92 ± 0.972	N/A	3.2032E-07 (p = 0.0019)

The incidence of postoperative complications, such as shivering, nausea, and vomiting, and the need for analgesics are presented in Table 6. None of the participants experienced shivering. Nausea and vomiting were observed in two participants in the remifentanil group, and the need for postoperative analgesics was recorded in four (10%) patients in the dexmedetomidine group and 32 (80%) patients in the remifentanil group.

**Table 6:** Postoperative complications: shivering, nausea and vomiting, and the need for analgesics.

Postoperative complications	Dexmedetomidine (n = 40)	Remifentanil (n = 40)
Shivering, n (%)	0	0
Nausea and vomiting, n (%)	0	2 (5%)
Need for analgesics, n (%)	4 (10%)	32 (80%)

The summarized results obtained from the preoperative and postoperative surveys of the participants regarding the quality of recovery are presented in Table 7. There was a small statistically significant difference between the two groups in the preoperative responses (p = 0.09) and a highly significant statistical difference in the postoperative responses (p < 0.05) in almost all responses.

**Table 7:** Postoperative recovery quality of dexmedetomidine/remifentanil expressed as QoR-15 scores. Data are presented as mean ± SD. QoR-15 = Quality of Recovery-15

QoR-15	Preoperative			Postoperative		
Questionnaire	Dexmedetomidine	Remifentanil	t-test (p-value)	Dexmedetomidine	Remifentanil	t-test (p-value)
Able to breathe easy	9.60 ± 0.52	9.6 ± 0.61	0.5 (0.6184)	7.13 ± 1.3	7.5 ± 2.56	2.46934E-06 (p = 0.015)
Able to enjoy food	9.61 ± 1.34	9.5 ± 0	0.296 (0.7680)	6.54 ± 1.3	9.05 ± 0	0.08513 (p = 0.93)
Feeling rested	9.55 ± 1.19	9.42 ± 0.30	0.161 (p = 0.87)	5.66 ± 1.95	7.875 ± 2.3	8.49847E-05 (p < 0.000)
Have had a good sleep	8.82 ± 1.53	9.15 ± 0.25	0.046 (p = 0.96)	5.94 ± 1.4	6.475 ± 1.78	2.68864E-12 (p = 0.008)
Able for personal hygiene	9.75 ± 0.89	9.55 ± 0.21	0.5 (p = 0.61)	5.47 ± 2.38	6.05 ± 1.004	5.85501E-09 (p < 0.00)
Communicate with family	8.35 ± 1.56	9.15 ± 0.19	1.7E-11 (p = 0.86)	8.59 ± 0.9	6.82 ± 0.93	8.46084E-06 (p < 0.00)
Need medical support	9.32 ± 0.87	9.6 ± 0.18	0.048 (p = 0.09)	8.35 ± 1.0	5.25 ± 0.88	2.12104E-18 (p = 0.037)
Able to return to work	7.8 ± 1.21	8.88 ± 0.17	3.8E-08 (p = 0.00)	4.47 ± 1.95	5.82 ± 0.84	4.08349E-07 (p = 0.0001)
Feeling comfortable	7.85 ± 1.13	9.02 ± 0.16	2.27 p = 0.025)	6.89 ± 1.2	5.52 ± 0.80	2.47362E-10 (p = 0.015)
Feeling of well-being	8.15 ± 1.38	8.37 ± 0.15	0.0007 (p = 0.9)	5.67 ± 1.67	5.625 ± 0.77	1.14829E-07 (p = 0.254)
Moderate pain	8.15 ± 1.37	9.37 ± 0.15	2.1E-06 (p = 0.03)	3.83 ± 1.06	5.55 ± 3.09	5.01562E-11 (p < 0.000)
Severe pain	9.2 ± 0.97	9.82 ± 0.14	0.0005 (p = 0.99)	6.08 ± 1.59	5.5 ± 0	1.23139E-17 (p = 0.22)
Nausea and vomiting	9.65 ± 0.69	9.67 ± 0.14	4.56 (p = 0.00)	8.94 ± 1.02	6.45 ± 1.54	3.10133E-11 (p = 0.002)
Worried and anxious	7.55 ± 2.08	7.72 ± 0.13	1.57 (p = 0.12)	6.81 ± 1.58	6.375 ± 1.26	0.00079284 (p = 0.99)
Feeling sad/depressed	9.25 ± 1.23	8.62 ± 0.13	0.01 (p = 0.99)	7.72 ± 1.11	5.9 ± 1.10	2.46934E-06 (p = 0.015)
Total	126.41 ± 7.6	137.4 ± 3.71	1.09 (p = 0.27)	92.72 ± 32	95.77 ± 32.7	0.08513915 (p = 093)
Preoperative: postoperative	Dexmedetomidine	Remifentanil	0.0007 (p = 0.9)	Dexmedetomidine	Remifentanil	N/A

To determine which postoperative factors played a key role in the quality of postoperative recovery, a comparison was made between QoR-15 and MAP, degree of postoperative sedation and pain, and respiratory variables. Pearson’s correlation coefficient was statistically calculated, and the results are shown in Table 8. In the dexmedetomidine group, a positive correlation was found between QoR-15 and RRS 2, the SatO_2_:EtCO_2_ ratio, and VAS 0 and VAS 1 with QoR-15, while in the remifentanil group, a positive correlation was observed between SAP:QoR-15, RRS 2:QoR-15, RRS 3:QoR-15, Sat:EtCO_2_, Sat:QoR-15, EtCO_2_:QoR-15, and VAS 0:QoR-15.

**Table 8:** Correlation coefficient (r) between postoperative MAP, sedation (RSS) and pain (VAS) on the quality of postoperative recovery. r = Pearson’s correlation coefficient; MAP = mean arterial pressure; VAS = Visual Analog Scale; RSS = Ramsay Sedation Scale; QoR-15 = Quality of Recovery-15

Variable	Dexmedetomidine (n = 40)	Remifentanil (n = 40)
MAP: QoR-15	r = -0.03933	r = 0.25036
RRS 0: QoR-15	r = -0.12268	r = -0.00967
RRS 1: QoR-15	r = -0.11723	r = -0.01855
RRS 2: QoR-15	r = 0.02012	r = 0.04089
RRS 3: QoR-15	r = -0.00341	r = 0.10229
VAS 0: QoR-15	r = 0.26969	r = -0.07177
VAS 1: QoR-15	r = 0.20386	r = -0.04049
VAS 2: QoR-15	r = -0.07855	r = -0.01441
VAS 3: QoR-15	r = -0.21731	r = -0.01855

## Discussion

The results obtained in this study confirmed the research goals. Dexmedetomidine is a potent analgesic and sedative agent, which, owing to its analgesic properties, rarely requires additional analgesics, as confirmed by this study, making it an ideal agent for use in soft tissue surgery [17]. However, as a sympatholytic agent, it reduces the release of norepinephrine from sympathetic nerve endings; therefore, its effect on the cardiovascular system is dose dependent. At low doses, the central action predominates, manifesting as hypotension and bradycardia, while at higher doses, the peripheral vasoconstrictive effect predominates, which increases vascular resistance during systole, causing hypertension and bradycardia. In this study, the perioperative hemodynamic and respiratory effects of dexmedetomidine and remifentanil were compared. When used at therapeutic doses, both caused a significant drop in systolic arterial pressure (MAP) (p < 0.005), which has also been described by other studies [18]. A significant difference was observed in respiratory effects. Dexmedetomidine does not cause respiratory depression, while remifentanil reduces the respiratory rate, which falls to the lower limit of normal values, as reported by other studies [19]. Perioperative blood saturation in both study groups remained within the normal range. Significant differences between the groups were noted in the preoperative values and five minutes after the start of the surgery (p < 0.005). Safe anesthesia was maintained for all participants in the study, with optimal perioperative hemodynamic and respiratory values.

When analyzing the postoperative values of inflammation markers (leukocytes) and stress markers (blood glucose level), the leukocyte count did not change compared to the preoperative values in both groups. This is explained by the short duration of the interventions and the type of surgical procedures, as well as the need for time for changes in leukocyte count to occur. In contrast, an increase in interleukin 6 (IL-6) was noted in the remifentanil group, but not in the dexmedetomidine group, indicating that IL-6 is a faster marker of inflammation, which confirms that dexmedetomidine has an anti-inflammatory effect. This is consistent with previous studies suggesting the anti-inflammatory properties of dexmedetomidine [20-23].

Preoperative and postoperative quality of recovery was assessed in all patients using the QoR-15 questionnaire, which showed a significant decrease in the immediate postoperative period in both groups. Postoperative complications after general anesthesia and procedural sedation have been intensively researched by many studies. Among the most common are postoperative nausea and vomiting (PONV), shivering, and postoperative pain. Shivering occurs in 20-70% of cases after general anesthesia and is a result of intraoperative cooling during long surgical interventions [24,25]. According to the study by de Boer et al., postoperative occurrence of PONV is associated with the use of opioids during anesthesia and sedation [26]. The results of this study showed that the use of dexmedetomidine and remifentanil in soft tissue surgery with relatively short duration did not cause shivering, while postoperative nausea was noted in two patients in the remifentanil group. This is explained by the opioid properties of the drug, which were successfully managed with the use of antiemetics. Huang et al. (2024) reported a dose-dependent initiation of PONV when using remifentanil [27], which explains the results of our study where a low dose of remifentanil was used.

Postoperative pain, measured using the VAS scale at four time points (T0-T3), showed significantly better analgesia immediately after awakening in patients treated with remifentanil (p < 0.005), which is a result of the analgesic effect of opioids. However, two hours after awakening, patients receiving remifentanil showed a higher level of pain compared to those treated with dexmedetomidine (4.425 ± 2.667 vs. 2.47 ± 0.62). This confirmed the prolonged analgesic effect of dexmedetomidine due to its direct central action on the LC [28].

The level of sedation in the postoperative period, measured using the RSS at four time points, showed a state of unconsciousness such as natural sleep with easy awakening in the dexmedetomidine group. However, a significantly greater sedation was noted in the remifentanil group immediately after the postoperative period and 60 minutes after awakening (p = 0.001225 and p = 0.000581, respectively). The sedative effects of dexmedetomidine are a result of the reduction of neural transmission at the level of the brainstem, leading to a state of unconsciousness like sleep and cooperativeness [29].

To determine which perioperative and postoperative factors influenced recovery during analgesic sedation with dexmedetomidine or remifentanil, a comparative analysis was conducted between the recovery outcomes (QoR-15) and factors such as MAP, blood oxygen saturation percentage, sedation level (RSS), and pain level (VAS). Statistical correlation analysis was performed to determine Pearson’s correlation coefficient. The analysis showed a positive correlation between Sat:EtCO_2_ and RSS 2 in both groups. In the dexmedetomidine group, a negative correlation was observed with MAP at T0, RSS 0, RSS 1, and RSS 3, as well as with VAS 2 and VAS 3. In the remifentanil group, a negative correlation was found with RSS 0 and RSS 1, as well as with all four time points for pain levels from VAS 0 to VAS 3. These findings highlight sedation level and pain level as key factors influencing the quality of postoperative recovery. Specifically, the stronger the pain and the higher the sedation level, the lower the QoR-15 score. This suggests that, for soft tissue surgery, the use of dexmedetomidine allows for same-day discharge after surgery with adequate postoperative observation (around four hours), while patients treated with remifentanil, due to the trend of increasing pain and sedation, should be discharged the following day.

Study limitations

A key limitation of this study was the change from its primary design, leading to the investigation of only the basic markers of inflammation and stress. Furthermore, the study enrolled a small sample, which may hinder the generalization of the results. Future research with larger cohorts and more extensive data will enhance the validity and applicability of our findings.

## Conclusions

Based on the results obtained in this study, dexmedetomidine offers a range of pharmacological advantages, providing dose-dependent sedation, sympatholysis, and anxiolysis without a significant respiratory depression. Its side effects are predictable and align with the pharmacological profile of alpha-2 adrenergic receptor agonists. The unique sedative action of dexmedetomidine promises several benefits for anesthesiology practice and can be safely used as a drug for analgesic sedation in short soft tissue surgical procedures. In this study, dexmedetomidine demonstrated a superior performance compared to remifentanil. Hence, in soft tissue surgery, dexmedetomidine ensures hemodynamic stability, shows protective anti-inflammatory and anti-stress effects, provides good postoperative analgesic effects, reduces recovery time, and protects the body from undesirable postoperative complications, such as shivering, severe pain, and PONV.
